# Genome-wide identification and expression analysis of the B-box transcription factor gene family in grapevine (*Vitis vinifera* L.)

**DOI:** 10.1186/s12864-021-07479-4

**Published:** 2021-03-29

**Authors:** Xiuming Zhang, Li Zhang, Miaomiao Ji, Yifei Wu, Songlin Zhang, Yanxun Zhu, Jin Yao, Zhi Li, Hua Gao, Xiping Wang

**Affiliations:** 1 State Key Laboratory of Crop Stress Biology in Arid Areas, College of Horticulture, Northwest A&F University, Yangling, 712100 Shaanxi China; 2 Key Laboratory of Horticultural Plant Biology and Germplasm Innovation in Northwest China, Ministry of Agriculture, Northwest A&F University, Yangling, 712100 Shaanxi China

**Keywords:** Grapevine, *BBX* family, Transcription factors, Expression profile

## Abstract

**Background:**

B-box (BBX) zinc-finger transcription factors play important roles in plant growth, development, and stress response. Although these proteins have been studied in model plants such as *Arabidopsis thaliana* or *Oryza sativa*, little is known about the evolutionary history or expression patterns of BBX proteins in grapevine (*Vitis vinifera* L.).

**Results:**

We identified a total of 25 *VviBBX* genes in the grapevine genome and named them according to the homology with Arabidopsis. These proteins were classified into five groups on the basis of their phylogenetic relationships, number of B-box domains, and presence or absence of a CCT domain or VP motif. BBX proteins within the same group showed similar exon-intron structures and were unevenly distributed in grapevine chromosomes. Synteny analyses suggested that only segmental duplication events contributed to the expansion of the *VviBBX* gene family in grapevine. The observed syntenic relationships between some *BBX* genes from grapevine and *Arabidopsis* suggest that they evolved from a common ancestor. Transcriptional analyses showed that the grapevine *BBX* genes were regulated distinctly in response to powdery mildew infection and various phytohormones. Moreover, the expression levels of a subset of *BBX* genes in ovules were much higher in seedless grapevine cultivars compared with seeded cultivars during ovule development, implying a potential role in seed abortion. Additionally, VviBBX8, VquBBX15a and VquBBX29b were all located in the nucleus and had transcriptional activity except for VquBBX29b.

**Conclusions:**

The results of this study establish the genome-wide analysis of the grapevine *BBX* family and provide a framework for understanding the biological roles of *BBX* genes in grapevine.

**Supplementary Information:**

The online version contains supplementary material available at 10.1186/s12864-021-07479-4.

## Background

Transcription factors (TFs) play varied and important roles in plant growth, development and biological responses [1]. Zinc-finger TFs are one of the most populous classes of TFs in plants, and can be classified into several families based on the number and location of characteristic amino acid sequence motifs [2]. The B-box (BBX) zinc-finger TFs contain one or two conserved domains of approximately 40 amino acids near the amino terminus, and may also contain a CCT (CONSTANS, CO-like and TOC1) domain and/or a valine-proline (VP) motif at the carboxyl terminus [3]. In *Arabidopsis thaliana* (Arabidopsis), *BBX* genes have been identified and classified into five subfamilies based on presence of these domains [4]. Additionally, it has been reported that the conserved B-box domain mediates protein-protein interactions, while the CCT domain functions in transcriptional regulation [5, 6].

BBX proteins participate in myriad biological processes in plants. In Arabidopsis, AtBBX1, AtBBX4, AtBBX7 and AtBBX32 regulate photoperiodic flowering [7–10]. Meanwhile, similar flowering roles have been reported in rice [11], barley [12], sorghum [13] and Chinese Cabbage [14]. Other studies have found that several AtBBX proteins participate in seedling photomorphogenesis through the HY5-COP1 regulatory module [15–17]. For instance, the HY5 transcription factor directly binds to a G-box *cis*-element present in the promoters of *AtBBX30* and *AtBBX31* and represses their expression, thus negatively regulating photomorphogenesis [18]. In rice, OsBBX14 promotes photomorphogenesis by directly binding the T/G-box *cis*-element of the *OsHY5L1* promoter under blue light conditions [19]. In pear, PpBBX16 (the homolog of AtBBX22) and PpHY5 jointly activate the expression of *PpMYB10* and other structural genes to positively regulate light-induced anthocyanin accumulation [20]. In apple, MdBBX20 integrates the influence of ultraviolet radiation and low temperature to promote the accumulation of anthocyanin [21]. In addition, BBX proteins have also been found to participate in response to environmental stress. For example, AtBBX31 promotes tolerance to UV-B radiation in Arabidopsis [22], and *CmBBX22* regulates leaf senescence in chrysanthemum [23]. Heterologous expression of apple *MdBBX10* in Arabidopsis enhances tolerance to salt [24]. In grapevine, *VvCOL* and *VvCOL1* (*VviBBX2* and *VviBBX5*, respectively) participate in flowering and bud dormancy [25].

Grapevine (*Vitis vinifera* L.) is one of the most economically important perennial fruit crops throughout the world. Grapes can be consumed fresh or dried, or can be processed into juice, wine, and jam. Seedless grapevine cultivars are particularly important, especially for fresh and dried fruit. However, worldwide production of both seeded and non-seeded grapes is increasingly limited by biotic and abiotic stresses. Cultivar improvement through traditional breeding and biotechnology is an exciting prospect, but options have been limited by the general lack of knowledge about key genes that mediate stress responses. Fortunately, the publication of a draft grapevine genome [26] has facilitated the identification of transcription factors. In this study, we identified members of the BBX family in grapevine from the draft genome sequence and gained insight into their potential function based on gene and protein structure, phylogeny, synteny, subcellular localization and transcriptional activity, as well as expression during ovule development, in response to pathogen challenge and various phytohormones. Taken together, this work will be helpful for future studies of *BBX* gene functions in grapevine.

## Results

### Identification and characterization of grapevine *BBX* genes

To identify *BBX* genes in the grapevine genome, we employed a Hidden Markov Model (HMM)-based approach and the amino acid sequence profile of the B-box-type zinc-finger domain (Pfam; PF00643). The resulting protein sequences were assessed for the presence of a B-box domain as defined by the Simple Modular Architecture Research Tool (SMART; http://smart.embl-heidelberg.de/) and the Conserved Domain Database (CDD; https://www.ncbi.nlm.nih.gov/Structure/cdd/cdd.shtml). This resulted in the identification of 25 putative *BBX* genes. For the sake of nomenclature and consistency, these were designated as *VviBBXs* (Table 1, Additional file 1: Text S1), based on the recently proposed grapevine nomenclature system [27]. The length of the encoded proteins ranged from 127 to 469 amino acids, and their predicted molecular mass ranged from ~ 14.3 to 50.9 kDa. The isoelectric points of the predicted proteins ranged from ~ 4.1 to 8.7 (Table 1).

**Table 1:** Detailed information of *VviBBX* gene family members in grapevine

Gene ID	VCost.v3 ID	CRIBI v2.1 ID	Locus ID	Accession no.	CDS (bp)	Protein (aa)	Position	MW (Da)	pI	Domains	Structural group	Subcellular localization
VviBBX2	Vitvi14g01296.t01	VIT_214s0083g00640.1	GSVIVT01036499001	XP_002282509.1	1176	391	chr14: 22695952–22698379 (+)	42699.57	5.77	2BBOX + CCT + VP motif	I	Nuclear
VviBBX5	Vitvi04g00665.t01	VIT_204s0008g07340.1	GSVIVT01036037001	XP_002263458.1	1044	347	chr4: 7669506–7671340 (−)	38001.35	6.24	2BBOX + CCT + VP motif	I	Nuclear
VviBBX6	Vitvi11g01309.t01	VIT_211s0052g01800.1	GSVIVT01029107001	XP_002277953.1	1086	361	chr11: 19920787–19922322 (+)	38993.62	6.75	2BBOX + CCT + VP motif	I	Nuclear
VviBBX7	Vitvi12g00757.t01	VIT_212s0057g01350.1	GSVIVT01030127001	XP_002264506.2	1248	415	chr12: 9692684–9699362 (−)	44982.85	4.76	2BBOX + CCT	II	Nuclear
VviBBX8	Vitvi10g00219.t01	VIT_200s0194g00070.1	GSVIVT01003473001	XP_002265377.2	1245	414	chr10: 2260168–2272241 (+)	45041.34	5.14	2BBOX + CCT	II	Nuclear
VviBBX9	Vitvi19g00408.t01	VIT_219s0014g05120.1	GSVIVT01014591001	XP_010644324.1	1113	370	chr19: 5406770–5409091 (−)	41137.3	6.33	2BBOX + CCT	II	Nuclear
VviBBX10	Vitvi12g00542.t01	VIT_212s0059g02500.1	GSVIVT01030628001	XP_019078853.1	1305	434	chr12: 7291808-7293953 (+)	47040.03	7.68	2BBOX + CCT	II	Nuclear/Extracellular
VviBBX11	Vitvi07g00252.t01	VIT_207s0104g01360.1	GSVIVT01010991001	XP_002268490.1	1185	394	chr7: 2762240–2765564 (−)	43918.32	6.22	2BBOX + CCT	II	Nuclear
VviBBX12a	Vitvi01g01729.t01	VIT_201s0146g00360.1	GSVIVT01000951001	XP_002263613.1	1410	469	chr1: 23236072–23239673 (−)	50856.79	5.89	2BBOX + CCT	II	Nuclear
VviBBX12b	Vitvi14g01487.t01	VIT_214s0068g01380.1	GSVIVT01033017001	XP_010660698.1	1350	449	chr14: 25084927–25088384 (+)	49441.06	6.09	2BBOX + CCT	II	Nuclear
VviBBX15a	Vitvi01g00288.t01	VIT_201s0011g03520.1	GSVIVT01011897001	XP_002282578.1	1299	432	chr1: 3190849–3193178 (−)	47784.66	5.40	1BBOX + CCT	III	Nuclear
VviBBX15b	Vitvi17g00328.t01	–	–	XP_002276181.1	1233	410	chr17: 3813174–3815238 (−)	45929.41	5.29	1BBOX + CCT	III	Nuclear/Cytoplasmic
VviBBX19a	Vitvi03g00049.t01	VIT_203s0038g00690.1	GSVIVT01024173001	XP_002267957.1	633	210	chr3: 594823–598603 (−)	23371.44	6.40	2BBOX	IV	Extracellular
VviBBX19b	Vitvi04g01423.t01	VIT_204s0023g03030.1	GSVIVT01018818001	RVW36633.1	555	184	chr4: 19619474–19622085 (−)	20366.2	6.87	2BBOX	IV	Extracellular
VviBBX21a	Vitvi03g00026.t01	VIT_203s0038g00340.1	GSVIVT01024204001	XP_002274649.1	909	302	chr3: 310821–312269 (+)	33371.52	7.79	2BBOX	IV	Nuclear
VviBBX21b	Vitvi18g01048.t01	VIT_218s0001g13520.1	GSVIVT01009821001	XP_002280716.1	912	303	chr18: 11546101–11548030 (+)	33157.15	6.65	2BBOX	IV	Nuclear
VviBBX22a	Vitvi18g02424.t01	VIT_218s0089g01280.1	GSVIVT01037095001	XP_019071822.1	618	205	chr18: 34402834–34404843 (+)	22812.56	5.05	2BBOX	IV	Nuclear
VviBBX22b	Vitvi19g00031.t01	VIT_219s0014g00350.1	GSVIVT01014097001	XP_002283666.1	882	293	chr19: 356889–371959 (+)	31682.56	4.87	2BBOX	IV	Extracellular
VviBBX25	Vitvi05g01519.t01	VIT_205s0102g00750.1	GSVIVT01010794001	XP_002268700.1	720	239	chr5: 23065597–23068639 (−)	26443.94	4.61	2BBOX	IV	Extracellular
VviBBX27	Vitvi01g00346.t01	VIT_201s0011g04240.1	GSVIVT01011821001	XP_002279997.2	1074	357	chr1: 3849170–3854088 (+)	39946.58	4.76	2BBOX	IV	Nuclear
VviBBX28	Vitvi10g02328.t01	VIT_200s0203g00210.1	–	RVW13823.1	639	212	chrUn: 11688677–11689572 (−)	23153.46	4.43	1BBOX	V	Nuclear
VviBBX29a	Vitvi12g02441.t01	VIT_212s0134g00400.1	GSVIVT01000440001	XP_002272924.1	900	299	chr12: 8048322–8050082 (+)	32306.61	4.06	1BBOX	V	Nuclear
VviBBX29b	Vitvi19g00322.t01	VIT_219s0014g03960.1	GSVIVT01014471001	XP_002284274.1	897	298	chr19: 4194453–4195989 (−)	32739.41	4.22	1BBOX	V	Nuclear
VviBBX30	Vitvi12g00543.t01	VIT_212s0059g02510.1	–	XP_010657357.1	384	127	chr12: 7296673–7297056 (−)	14258.41	7.71	1BBOX	V	Extracellular
VviBBX32	Vitvi09g01361.t01	VIT_209s0054g00530.1	–	RVX03703.1	783	260	chr9: 21166392–21167745 (+)	28155.08	8.67	1BBOX	V	Nuclear

Abbreviations: CDS, coding sequence; aa, amino acid; chr, chromosome; Un, unknown chromosome; MW, molecular weight; pI, isoelectric point

### Phylogeny and conserved domains of the grapevine BBX proteins

**Fig. 1:**
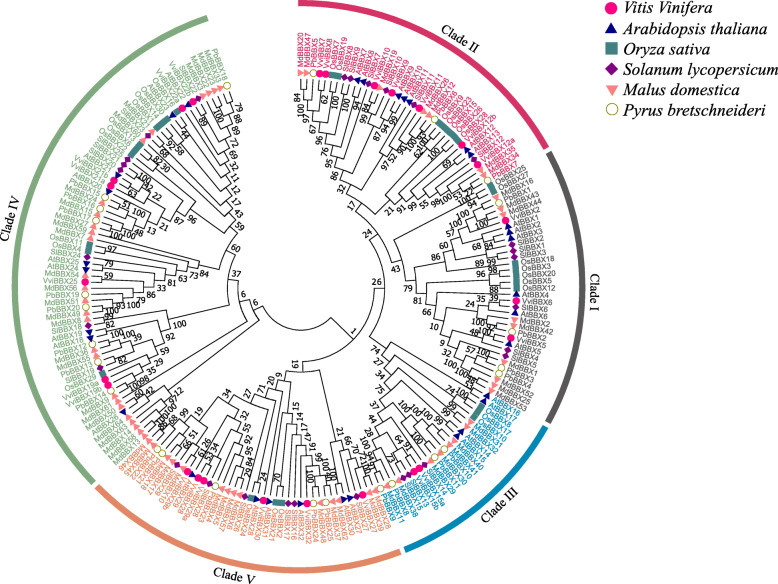
Phylogenetic analysis of BBX proteins from grapevine, Arabidopsis, tomato, apple, pear and rice. The tree was divided into five clades, which are marked by different colors and named as Clade I, II, III, IV and V. The bootstrap values are indicated at each node

To analyze the evolutionary relationship and potential functional divergence of the *VviBBX* gene family, a total of 205 BBX proteins, including 32 from Arabidopsis, 29 from tomato, 30 from rice, 64 from apple and 25 from pear, were used to construct a phylogenetic tree (Fig. 1, Additional file 2: Text S2). This resolved the grapevine BBX proteins into five clades which mostly corresponded to their assigned structural groups (Table 1, Fig. 3a). However, an exception was found in the above clades. The VviBBX27 protein was presumptively phylogenetically in clade IV based on the structure group, but it was located in phylogenetic clade V (Fig. 1). As shown in the phylogeny tree, it is evident that *BBX* genes of the woody plants (grapevine, apple and pear) clustered together. And most of the grapevine BBXs also clustered together with proteins from Arabidopsis and tomato, instead of rice, consistent with the closer relationship of grapevine to the two eudicots.

**Fig. 2:**
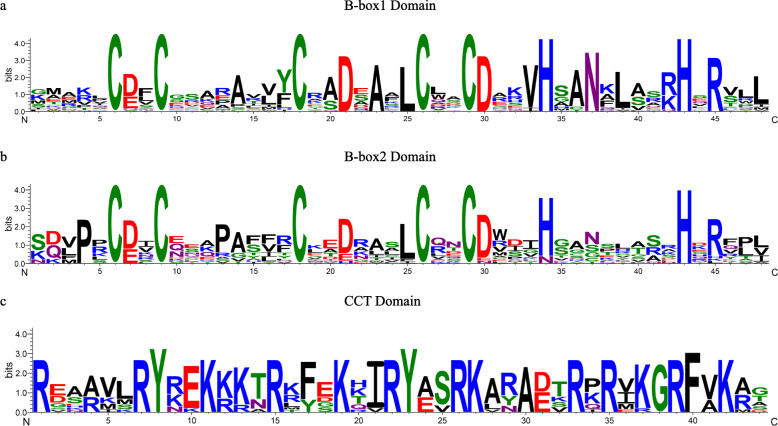
Amino acid sequence conservation within the B-box and CCT domains of grapevine BBX proteins. (a), (b) and (c) represent the amino acid sequence alignment of the B-box1, B-box2 and CCT domain, respectively. The x axis indicates the amino acids present at each position, and the y-axis and height of each letter indicate the degree of conservation of each residue across all proteins

The conserved sequences of the B-box1 and B-box2 zinc finger domains were C-X_2_-C-X_7–8_-C-X_2_-D-X-A-X-L-C-X_2_-C-D-X_3_-H-X_2_-N-X_4_-H and C-X_2_-C-X_8_-C-X_7_-C-X_2_-C-X_4_-H(N)-X_6–8_-H, respectively. In addition, the CCT domain of twelve of the grapevine proteins with the form of R-X_5_-R-Y-X_2_-K-X_3_-R-X_3_-K-X_2_-R-Y-X_2_-R-K-X_2_-A-X_2_-R-X-R-X_2_-G-R-F-X-K was highly conserved. A graphical representation of amino acid conservation with these motifs is shown in Fig. 2. Alignment of the protein sequences revealed that the B-box1 domain was more conserved than the B-box2 as a result of five absolutely conserved amino acid residues (two Asps, Ala, Leu and Asn) in all B-box1 domain (Additional file 3: Fig. S1).

Based on amino acid sequence conservation, number of B-box domains, and the presence or absence of the CCT domain, the 25 grapevine BBX proteins fell into five distinct structural classes (Table 1), which is consistent with previous results in Arabidopsis [4]. Group I, comprising three of the proteins, contained two B-box domains and one CCT domain. The seven representatives of Group II also contained two B-box domains and a CCT domain, but were distinguished from Group I based on the absence of the highly conserved amino acid sequence (SANPLARR) in the B-box2 domain and VP motif amino-terminal to the CCT domain seen in Group I proteins (Additional file 4: Fig. S2). Group III, comprising two proteins, contained one B-box domain and one CCT domain. The eight members of Group IV contained two B-box domains, while Group V proteins (five members) had only one B-box domain.

### Analysis of conserved protein motifs and exon-intron structure of *VviBBX* genes

**Fig. 3:**
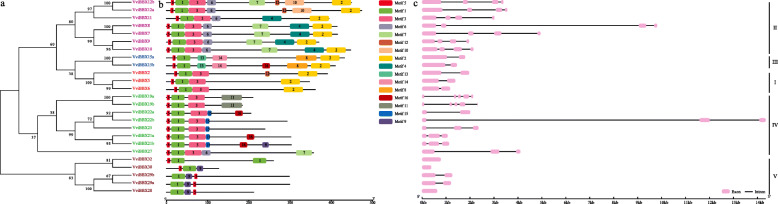
Characterization of grapevine *BBX* genes. **a** Phylogenetic analysis of BBX proteins in grapevine. **b** Distribution of conserved motifs identified in the 25 VviBBX proteins. Each motif is represented by a number in a colored box. Detailed sequence information for each motif is shown in Additional file 5: Table S1. **c** Exon-intron structure of grapevine *BBX* genes. Exons are represented by pink boxes and black lines connecting two exons represent an intron. The Roman numerals (I-V) indicate the five structural groups

To gain additional insight into the conservation and diversification of the grapevine *BBX* gene family, we analyzed the conserved protein motifs encoded by the genes, as well as exon-intron structures (Fig. 3). Sixteen conserved motifs were identified (Fig. 3b), with four corresponding to B-box1 (Motifs 1/5), B-box2 (Motif 3), and CCT (Motif 2). Interestingly, we observed that Motifs 8, 13, and 14 were present only in Group III, which might contribute to the functional divergence of *BBX* genes. Motifs 6 and 7 were seen in all members of Group II, but also in VviBBX27 in Group IV, suggesting that *VviBBX27* may have evolved from a Group II gene. The motif sequences and logos are listed in Additional file 5: Table S1. Additionally, *VviBBX22b* was the longest *BBX* gene (14.3 Kb). We also found that three genes in Group V (*VviBBX28*, *VviBBX30* and *VviBBX32*) had a single exon, while all others carried between two and five exons. Moreover, all the genes in Groups I, II and III contained three, four and two exons, respectively (Fig. 3c).

### Chromosomal distribution and synteny analysis among *VviBBX* genes

**Fig. 4:**
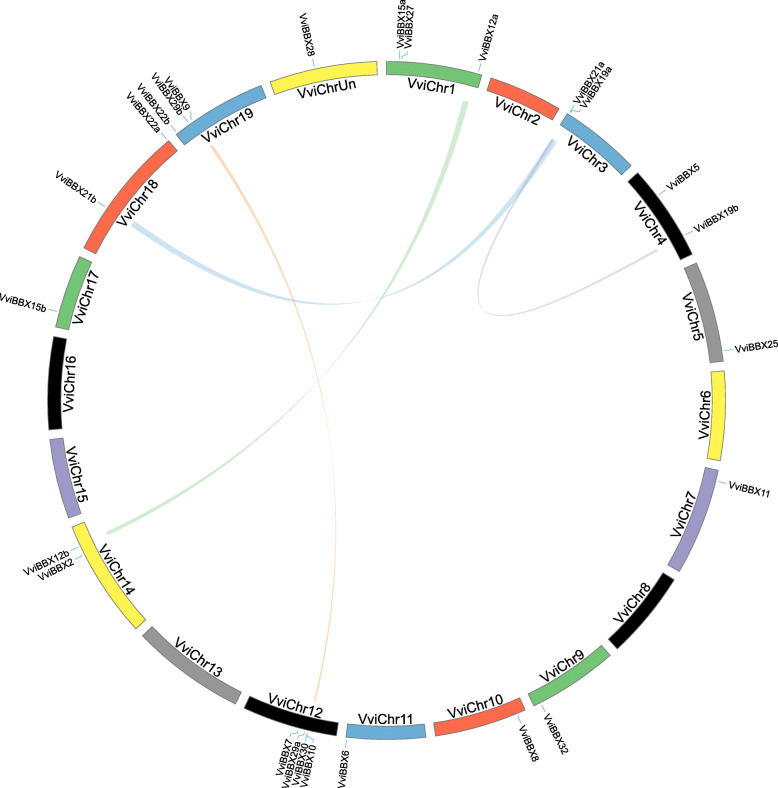
Distribution and synteny analysis of *VviBBX* genes on grapevine chromosomes. The approximate chromosomal locations of the *BBX* genes are indicated on the periphery. The colored lines linking genes from different chromosomes denote segmental duplication events

Based on their annotated genomic locations, the 25 *VviBBX* genes were found to be widely distributed among the grapevine chromosomes (Fig. 4). Chromosome 12 contained the most *VviBBX* genes (four), whereas Chromosomes 1 and 19 both possessed three genes, Chromosomes 3, 4, 14, and 18 had two *VviBBX* genes, and Chromosomes 5, 7, 9, 10, 11, and 17 had only one gene. The chromosomal location of *VviBBX28* was on the chromosome Unknown.

**Fig. 5:**
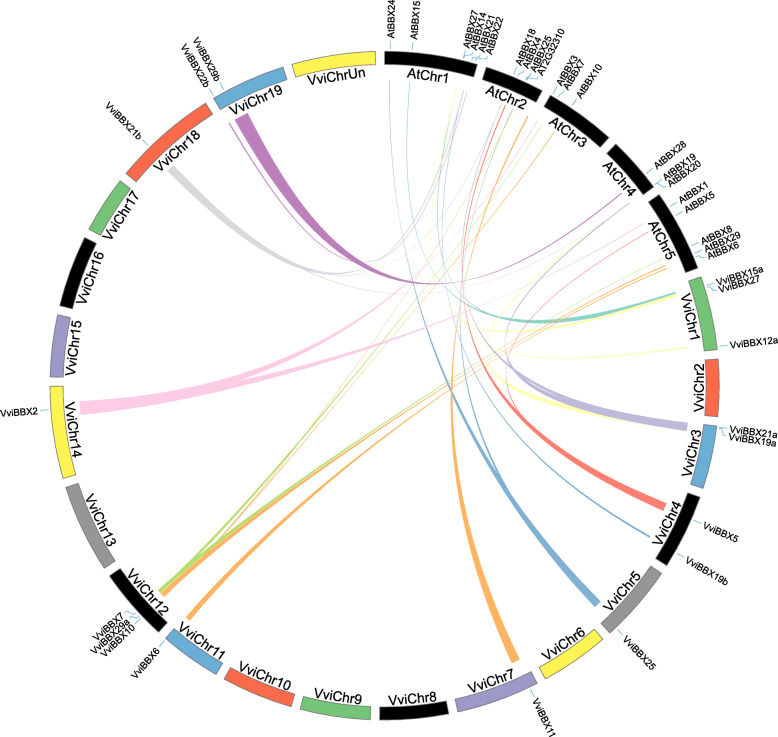
Synteny analysis of *BBX* genes between grapevine and Arabidopsis. The chromosomes of grapevine and Arabidopsis are arranged as a circle. Syntenic occurrences of *BBX* genes are represented by colored lines

Segmental duplications and tandem duplications contribute to the evolution of gene families [28]. According to Fig. 4 and Additional file 6: Table S2, four segmental duplication events have occurred: *VviBBX9* to *VviBBX10, VviBBX12a* to *VviBBX12b, VviBBX19a* to *VviBBX19b,* and *VviBBX21a* to *VviBBX21b*. However, no tandem duplication was observed according to the foregoing descriptions of Holub [29], and thus only segmental duplication seems to have taken part in the evolution of the grapevine *BBX* gene family. To gain insight into the evolutionary relationship between *VviBBX* and *AtBBX* genes, we analyzed genomic synteny. A total of 26 gene pairs, comprising 17 *VviBBXs* and 23 *AtBBXs*, were identified (Fig. 5, Additional file 7: Table S3). Among those, we found nine orthologous pairs, and also identified eight orthologous gene pairs with one grapevine gene corresponding to multiple *Arabidopsis* genes. We noted that AT2G32310 was not included in the *Arabidopsis* BBX family, but contained a CCT domain which was also found in VviBBX10. Finally, three orthologous gene pairs where multiple grapevine genes corresponded to a single *Arabidopsis* gene were found (Additional file 7: Table S3). In brief, these syntenic relationships suggest that about two-thirds of the *BBX* genes appeared before the divergence of grapevine and Arabidopsis.

To investigate potential selective pressure for *VviBBX* gene duplication events, we calculated the nonsynonymous (Ka) and synonymous (Ks) substitution rates. Between grapevine and Arabidopsis, or grapevine alone, all segmentally duplicated gene pairs showed Ka/Ks ratios of < 1, suggesting that they had evolved primarily under purifying selection. The divergence time of the segmental duplication event was calculated as between ~ 77 and 110 million years ago (Mya) in grapevine alone (Additional file 6: Table S2), and between ~ 102 and 349 Mya, with an average of 178.8 Mya, in grapevine and Arabidopsis (Additional file 7: Table S3).

### *VviBBXs* gene expression profiles in response to *E. necator* inoculation and hormone treatments

**Fig. 6:**
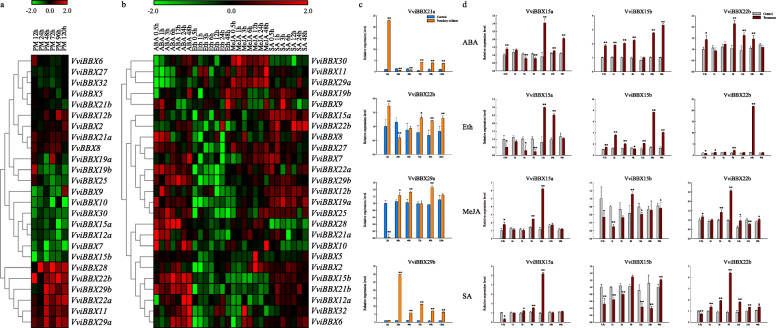
Expression profiles of 25 *VviBBX* genes following *E. necator* inoculation and various hormone treatments in grapevine. **a** Semi-quantitative RT-PCR expression analysis after *E. necator* inoculation and **b** under various hormone treatments (ABA: abscisic acid, Eth: ethylene, MeJA: methyl jasmonate and SA: salicylic acid) in ‘Shang-24’. Transcripts were normalized to the expression of the *ACTIN1* gene and *EF1-α* gene. **c** Quantitative RT-PCR analysis of expression of selected *VviBBX* genes after *E. necator* inoculation and **d** under various hormone treatments. The grapevine *ACTIN1* gene was used as an internal control to normalize expression levels. Mean values and standard deviations (SDs) are indicated by error bars. Asterisks indicate significance of the indicated differences in gene expression according to the *t*-test (*P< 0.05, **P< 0.01)

To help identify a possible function of the *VviBBX* genes in response to powdery mildew, we inoculated healthy plants of the powdery-mildew resistant genotype ‘Shang-24’ with *Erysiphe necator*, the causative agent of grapevine powdery mildew, and monitored the expression of the 25 *VviBBX* genes by semi-quantitative RT-PCR (Real-time polymerase chain reaction). Within 12 h after inoculation, the expression levels of ten genes (*VviBBX2*, *VviBBX8*, *VviBBX11*, *VviBBX12b*, *VviBBX21a*, *VviBBX22a*, *VviBBX22b*, *VviBBX28*, *VviBBX29a* and *VviBBX29b*) were up-regulated, while those of seven others (*VviBBX7*, *VviBBX9*, *VviBBX10, VviBBX12a*, *VviBBX15a*, *VviBBX15b* and *VviBBX30*) were down-regulated (Fig. 6a). Expression of *VviBBX6, VviBBX19b*, *VviBBX21a* and *VviBBX25* peaked at 12 h post-inoculation, whereas *VviBBX6* and *VviBBX19b* decreased sharply at 24 h and remained relatively stabile for the remainder of the measurement period.

Plant hormones, such as abscisic acid (ABA), ethylene (Eth), methyl jasmonate (MeJA), and salicylic acid (SA), play important roles in regulating developmental processes and signaling networks involved in plant responses to biotic and abiotic stresses [30]. In this study, we evaluated the transcriptional response of the 25 *BBX* genes in plants exposed to these hormones (Fig. 6b). In plants treated with ABA, *VviBBX15b*, *VviBBX21b*, *VviBBX22a*, *VviBBX28* and *VviBBX29b* were up-regulated to various degrees, while *VviBBX29a* was down-regulated. *VviBBX22b* transcript levels decreased slightly at the first three sampling times after ABA treatment, but then increased. In plants treated with ethylene, most *VviBBX* genes were down-regulated, while *VviBBX19b* and *VviBBX32* showed decreased expression at least at 12 and 24 h after treatment. After treatment with MeJA, three genes (*VviBBX27*, *VviBBX29a* and *VviBBX30*) were up-regulated, while four genes (*VviBBX12a*, *VviBBX15b*, *VviBBX21b* and *VviBBX22b*) were down-regulated. Interestingly, two genes, *VviBBX2* and *VviBBX15a*, showed an obvious decrease in expression at the early stages and increase in expression at the later stages. After treatment with SA, eight genes (*VviBBX2*, *VviBBX12b*, *VviBBX15a*, *VviBBX15b*, *VviBBX19a*, *VviBBX19b*, *VviBBX21b* and *VviBBX22b*) were up-regulated, and two genes (*VviBBX28* and *VviBBX30*) were down-regulated. Expression of *VviBBX21b* peaked 1 h after treatment, whereas that of both *VviBBX19a* and *VviBBX19b* peaked 3 h after treatment. These transcriptional responses show that the grapevine *BBX* genes are regulated by multiple phytohormones. To support the results of the semi-quantitative RT-PCR analyses, expression of six, randomly-selected *VviBBX* genes was determined using quantitative RT-PCR (Fig. 6c, d), and results of both analysis approaches were generally consistent.

### Expression analysis of *VviBBX* genes during ovule development

To insight into the potential functions of *VviBBX* genes during grapevine development, the gene expression atlas contained 54 various organs and tissues at different developmental stages was performed (Additional file 8: Fig. S3, Additional file 9: Table S4) based on the GEO DataSets (GSE36128) [31]. We noted most of the *VviBBX* genes showed different levels of expression in all organs and tissues. For example, *VviBBX6* and *VviBBX11* were high expressed relatively ubiquitously. *VviBBX10* showed higher expression level in stamen and pollen than other tissues. *VviBBX22b* exhibited lowest levels of expression in senescencing leaf (Additional file 8: Fig. S3). These results indicated the functional diversification of *VviBBX* genes during grapevine growth and development.

**Fig. 7:**
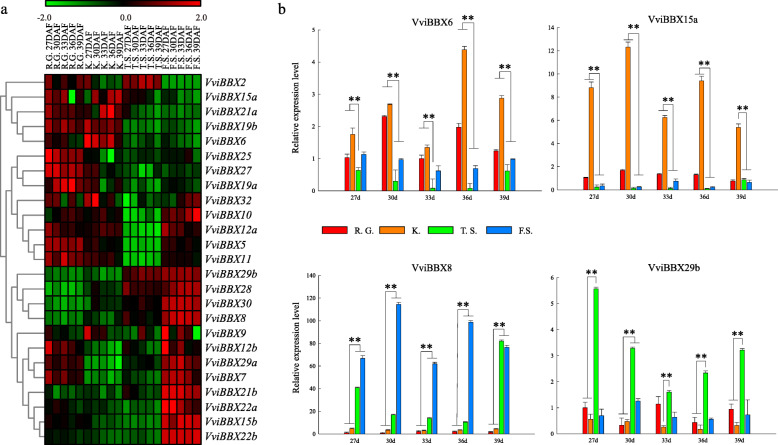
Expression analysis of 25 *VviBBX* genes in two seeded cultivars, ‘Red Globe’ (R.G.) and ‘Kyoho’ (K.), and two seedless cultivars, ‘Thompson Seedless’ (T.S.) and ‘Flame Seedless’ (F.S.). **a** Semi-quantitative RT-PCR expression analysis. Transcripts were normalized to the expression of the *ACTIN1* gene and *EF1-α* gene. **b** Quantitative RT-PCR analysis of expression of selected *VviBBX* genes. The grapevine *ACTIN1* gene was used as an internal control to normalize expression levels. Mean values and standard deviations (SDs) are indicated by error bars. Asterisks indicate significance of the indicated differences in gene expression according to the *t*-test (*P< 0.05, **P< 0.01)

Additionally, previous transcriptome analyses of seed development in grapevine hybrids also suggested that *VviBBX* transcription factors might be involved in seed size [32]. Hence, we analyzed the expression of the genes in two seeded cultivars (‘Red Globe’ and ‘Kyoho’) and two seedless cultivars (‘Thompson Seedless’ and ‘Flame Seedless’), at 27, 30, 33, 36 and 39 days after flowering (DAF) (Fig. 7a) to investigate a potential function for grapevine *BBX* genes in ovule development. We noticed that most *VviBBX* genes were expressed differentially according to cultivar, suggesting a potential role in seed development or abortion. Four genes (*VviBBX8*, *VviBBX28*, *VviBBX29b* and *VviBBX30*) exhibited much higher expression levels in seedless cultivars than seeded cultivars. In particular, *VviBBX8* showed a hundred-fold higher expression in seedless cultivars. In contrast, higher expression levels in seeded cultivars relative to seedless cultivars were observed for eight genes (*VviBBX5*, *VviBBX6 VviBBX10*, *VviBBX11*, *VviBBX12a*, *VviBBX15a*, *VviBBX19b* and *VviBBX21a*), suggesting that they might function in normal development of the ovule. Expression of four randomly selected genes was assessed by quantitative RT-PCR (Fig. 7b), and results were generally consistent with those obtained from semi-quantitative RT-PCR.

### Subcellular localization and transcriptional activity of the three BBX proteins

**Fig. 8:**
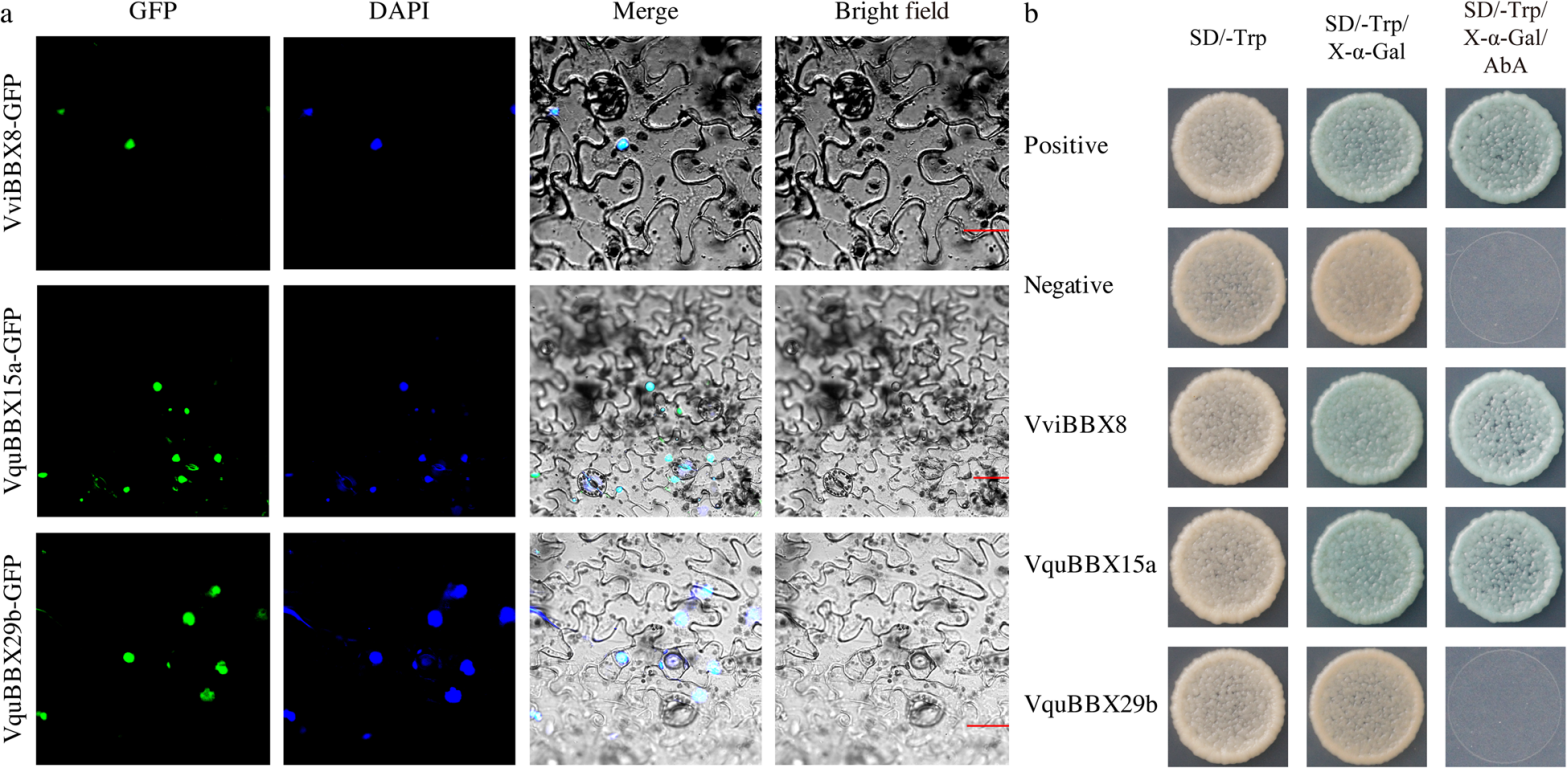
Subcellular localization and transcriptional activity analysis of the three *BBX* genes. **a** Subcellular localization of three GFP-fused grapevine BBX proteins in tobacco leaves. Bar = 20 μm. **b** Transactivation experiments with VviBBX8, VquBBX15a and VquBBX29b in yeast

As shown in Table 1, subcellular localization software predicted that approximately 80% BBX proteins were located at the nucleus. To verify the predicted localization pattern of BBX proteins in cells, three genes with one in *V. vinifera* cultivars ‘Thompson Seedless’ (*VviBBX8*) and two from Chinese wild *V. quinquangularis* accession ‘Shang-24’ (*VquBBX15a* and *VquBBX29b*), which strongly responded to powdery mildew, hormones and/or ovule development, were cloned for subcellular localization in tobacco leaves (Additional file 10: Fig. S4). The green fluorescence signals from VviBBX8-GFP, VquBBX15a-GFP and VquBBX29b-GFP fusion proteins were all observed specifically in the nucleus of tobacco leaves, suggesting that the three fusion proteins were localized in the nucleus (Fig. 8a), and these were consistent with the prediction results (Table 1). Using the Yeast Two-Hybrid System (Y2H), the transcriptional activities of BBX proteins were also analysed. Yeast cells transformed with the positive control and negative control all grew well on SD/−Trp (Lacking tryptophan) and SD/−Trp/X-α-Gal (5-Bromo-4-chloro-3-indolyl-α-Dgalactopyranoside). Positive blue colonies of yeast cells transformed with pGBKT7-VviBBX8 and pGBKT7-VquBBX15a were observed on SD/−Trp/X-α-Gal/AbA (Aureobasidin A), while yeast cells transformed with pGBKT7-VquBBX29b did not survive, suggesting that the VviBBX8 and VquBBX15a had transcriptional activity and VquBBX29b possessed no activation ability in yeast (Fig. 8).

## Discussion

In this investigation, 25 grapevine *BBX* genes were systematically identified and characterized using bioinformatic approaches. Detailed information about these genes, including gene ID, accession numbers, structural group classification, and physiological and biochemical properties of the encoded protein are given in Table 1. The number of *BBX* family genes was the same as found in pear [33] and was less than in Arabidopsis [4], rice [34], tomato [35], potato [36], apple [37] and maize [38], but higher than eight other plant species [38, 39]. We also found that the largest number of BBX members in a plant was 64 from apple, while the least was 19 in millet (Additional file 11: Fig. S5). The remarkable variation in gene number among plants, although potentially attributable to unfinished genome sequence, could reflect species-specific duplications or deletions during evolution. The genes were named *VviBBXs* (Table 1) based on the nomenclature rules and mapped to specific chromosomes according to their annotated genomic location (Fig. 4).

We constructed logos alignment for the two conserved B-box domains and CCT domain as shown in Fig. 2. The distribution of conserved amino acid residues between the B-box1 domain and B-box2 domain was similar, but not identical. The five Cys residues and two His residues were conserved to a greater extent than the remainder of the amino acid residues in the B-box1 domain. However, the first His residue following the second Cys-X-X-Cys was replaced by Asn in the B-box2 domain (*VviBBX9* and *VviBBX10*). This variation is not seen in Arabidopsis [4], rice [34], tomato [35] or apple [37] and might bring about a new function for these two genes. The 32 Arabidopsis *BBX* genes were unambiguously classified into five structural groups [4], and BBX members from other plants, including grapevine in this study, can be assigned into five groups (Table 1, Fig. 3a). However, the numerosity of each group was different in different plants. For instance, the numbers of BBX proteins with two B-box domains plus a CCT domain, one B-box domain plus a CCT domain, two B-box domains and only one B-box domain were 13, 4, 8 and 7 in Arabidopsis [4], 12, 5, 18 and 29 in apple [37], and 10, 2, 8 and 5 in grapevine (Additional file 11: Fig. S5). We carried out a phylogenetic analysis of BBX protein sequences from grapevine and five other plants. The 25 BBXs in grapevine were classified into five clades, mostly corresponding with the structural groups except for VviBBX27 (Table 1, Fig. 1, Fig. 3a). And some exceptions were also seen in tomato [35], potato [36] and apple [37]. For example, apple MdBBX7 and MdBBX59 with only one B-box domain were presumptively classified to Clade V, but they were phylogenetically in Clade IV, which contained two B-box domains [37].

We analyzed the conserved motifs in the grapevine BBX family genes by MEME, and only 16 motifs were authenticated with E-value < 0.05. The majority of the VviBBX proteins in the same group showed similar motif distribution, but exceptions were found (Fig. 3b). For example, Motif 8, Motif 13 and Motif 14 were found only in Group III, whereas Motif 4, Motif 6 and Motif 7 were mostly specific to Group II. Motif 10 was found only in VviBBX12a and VviBBX12b, the two genes of Group II. These results suggested that BBX proteins might have some unique functions. A previous study reported that exon-intron structure could be used to support phylogenic relationships in a gene family [40]. We found that the number of exons varied from 1 to 5, and most exons showed conserved positions among the 25 *VviBBX* genes. We also found that three genes (*VviBBX28*, *VviBBX30* and *VviBBX32*) in Group V, accounting for 12% of the entire family, had no introns, which was the same percentage observed in the pear *BBX* family [33]. As shown in Fig. 3, *VviBBX* genes containing a similar exon-intron structure clustered together in the phylogenetic tree. Indeed, we observed four gene pairs (*VviBBX9*/*VviBBX10*, *VviBBX12a*/*VviBBX12b*, *VviBBX19a*/*VviBBX19b* and *VviBBX21a*/*VviBBX21b*) possessing the same number of exons with nearly the same exon lengths (Fig. 3c), suggesting that they might have been generated from segmental or tandem duplication, which is supported by our synteny analysis (Fig. 4). However, exon-intron gain or loss, which contributes to expansion and diversification of gene families [41], was observed within the same *VviBBX* gene group. For example, *VviBBX22b* had three exons, while the paralogous gene *VviBBX22a* contained only two exons (Fig. 3c), suggesting that *VviBBX22a* might have lost one exon during evolution. A similar observation was reported within the five members of the grapevine *bHLH* IX subfamily, *VvbHLH103*-*VvbHLH107*. *VvbHLH103* contained five exons, while the other four members contained six, suggesting *VvbHLH103* might also lost one of its exons in the course of evolution [42].

Segmental and tandem genomic duplication are important driving forces in gene family expansion [28]. Duplicated genes generally undergo selection, including purifying selection, positive selection or neutral selection, to adapt to the unstable environmental conditions during the long period of evolution. In the present study, four segmentally duplicated gene pairs were found on grapevine Chromosomes 1, 3, 4, 12, 14, 18 and 19, whereas no tandemly duplicated genes were identified (Fig. 4). This result is similar to those from studies of *BBX* gene family evolution in pear [33], rice [34], tomato [35], maize, sorghum, stiff brome and millet [38]. Moreover, the segmentally duplicated gene pairs, (e.g., *VviBBX21a*/*VviBBX21b*) comprised the same group and exhibited similar exon-intron structures and motifs (Fig. 3b, c). A previous study in rice also showed that *OsBBX* genes likely resulting from segmental duplication were part of the same group [34]. These observations demonstrate that segmental duplications contributed to expansion of the *BBX* gene family in grapevine. We also identified 26 orthologous *BBX* gene pairs resulting from segmental duplications between grapevine and *Arabidopsis*, suggesting that they might have a common ancestor, and therefore, similar functions. For example, expression of *AtBBX5* was shown to be strongly induced by ABA, leading to enhanced abiotic stress tolerance [43]. In this study, we found that the orthologous *VviBBX5* is expressed to relatively high levels in ABA-treated plants (Fig. 6b). This implicates *VviBBX5* in abiotic stress responses in grapevine.

To date, the functions of BBXs have been widely reported, including photoperiodic regulation of flowering, shade avoidance, seedling photomorphogenesis, anthocyanin accumulation and abiotic stress. For example, in chrysanthemum, *CmBBX24* was shown to enhance drought and low temperature tolerance by modulating gibberellin biosynthesis [44]. A Group IV BBX protein in *Arabidopsis*, *AtBBX21*, functions in ABA signaling [45], and is a positive regulator of photomorphogenesis [17]. Our analyses of grapevine *BBX* gene expression in response to powdery mildew infection and phytohormone treatments (Fig. 6a, b) showed that these genes are diversely regulated. For instance, *VviBBX29b* was strongly induced after upon powdery mildew infection, while *VviBBX15a* was strongly induced and *VviBBX15b* was slightly repressed in response to MeJA treatment (Fig. 6c, d). These results provide evidence that BBX proteins may participate in response to biotic stress and hormonal signal transduction pathways.

In the present study, the expression levels of most *VviBBX* genes peaked 27–33 days after full bloom in seedless grapevine varieties (Fig. 7a). Similarly, some previous studies had reported that ovule abortion in stenopermocarpic seedless cultivars takes place at about this time [32, 46]. The expression patterns of *VviBBX8* and *VviBBX29b* were significantly up-regulated in nearly all ovule developmental stages in seedless grapevine cultivars, relative to seeded varieties. However, *VviBBX6* and *VviBBX15a* were significantly up-regulated in seeded grapevine cultivars (Fig. 7b)*.* Taken together, the difference in expression levels of *VviBBX* genes between the seeded and seedless cultivars suggests that they might be involved in ovule abortion, and also have a function in normal seed development.

AtBBX14 and AtBBX15 has been shown to participate in various signaling pathways to uniformize plant growth and development in *Arabidopsis* [15]. In this study, *VquBBX15a*, the same clade of *AtBBX14* and *AtBBX15*, has the representative features of the BBX family, which was located in the nucleus (Fig. 8a) and had transcriptional activity (Fig. 8b). These results indicate that VquBBX15a functions as a transcriptional activator of diverse downstream genes. Furthermore, we found that VquBBX29b possessed no activation ability in yeast, maybe it needs some required modification or assistance of other proteins. Definitely, more experiments are needed to confirm this suppose in the future.

## Conclusions

In this study, 25 *VviBBX* genes were identified based on the latest version of grapevine genome annotation. And we also carried out a comprehensive analysis of the *BBX* gene family, including phylogeny, conserved domain, motif compositions, exon-intron configurations, chromosomal distributions, genomic synteny and expression pattern analysis. The transcription of *VviBBX* in response to powdery mildew infection, various hormonal treatments and during seed development indicated that *VviBBX* genes might participate in the corresponding signal transduction pathways and seed abortion. Additionally, the subcellular location and transcriptional activity assays of three BBX members were verified, suggesting that BBX proteins might activate the expression of various downstream genes in nucleus. Taken together, genome-wide analysis of the *VviBBX* family will provide a fundamental basis for further research on the functions of *BBX* genes in grapevine.

## Methods

### Identification and annotation of *BBX* genes in the grapevine genome

To identify potential *BBX* genes in grapevine, the new grapevine reference genome assembly (12X.v2) and its VCost.v3 gene annotation were downloaded from URIG website (https://urgi.versailles.inra.fr/Species/Vitis/Annotations) [47]. The Hidden Markov Model (HMM) profile for the B-box-type zinc finger domain (PF00643) obtained from Pfam (http://pfam.xfam.org/family/PF00643) [48] was used to identify putative *BBX* genes in the grapevine genome using HMMER3.0 [49]. And the Expect (e) value cutoff was 0.01. The CRIBI v2.1 ID and Locus ID were obtained from the Phytozome v13 database (https://phytozome-next.jgi.doe.gov) [50] and Grape Genome Browser (12X) database (https://www.genoscope.cns.fr/vitis/) [26], respectively. The presence of a B-box domain was checked manually using SMART (http://smart.embl-heidelberg.de) [51] and the Conserved Domain Database (https://www.ncbi.nlm.nih.gov/Structure/cdd/wrpsb.cgi) [52]. The predicted mass and charge of the BBX proteins were calculated using the ProtParam tool (http://web.expasy.org/protparam/) [53].

### Phylogenetic and conserved domain alignments analysis

Amino acid sequences of the B-box and CCT domains were aligned with DNAMAN (Version 7.0.2, Lynnon Biosoft), and sequence logos were created using Weblogo 3 (http://weblogo.threeplusone.com) [54]. The Muscle module within the MEGA 7.0 software package [55] was used to align sequence of full length proteins, and phylogenetic trees were constructed by utilizing the Neighbor-Joining (NJ) approach with 1000 bootstrap replications, and the following parameters: Poisson model, uniform rates, same (homogeneous), and pairwise deletion. BBX protein sequences from *Arabidopsis thaliana* (*AtBBX*) [4], *Pyrus bretschneideri* (*PbBBX*) [33], *Oryza sativa* (*OsBBX*) [34], *Solanum lycopersicum* (*SlBBX*) [35] and *Malus domestica* (*MdBBX*) [37] were downloaded from genome databases maintained for each species.

### Analysis of exon-intron structure and conserved motifs

Exon-intron structures of the confirmed *BBX* genes were determined according to the alignments of their coding sequences and genomic full-length sequences in the Grapevine Genome (12X) database (https://www.genoscope.cns.fr/vitis/) [26]. The diagrams of exon-intron structures were generated using the online program Gene Structure Display Server 2.0 (http://gsds.cbi.pku.edu.cn) [56]. Conserved motifs of all BBX proteins were identified using the online MEME analysis tool (http://meme-suite.org/tools/meme) [57] with the maximum number of motifs being set at 16, and other default parameters. Only motifs with E-value < 0.05 were present. TBtools software was used to draw the map of the conserved motif [58].

### Chromosomal localization and synteny analysis

The chromosomal locations of each *VviBBX* gene were identified according to physical location information from the latest version of grapevine genome annotation. Syntenic blocks for the grapevine *BBX* genes, as well as between grapevine and Arabidopsis, were identified and analyzed using the MCScanX software [59]. Synteny analysis and chromosomal location diagrams were generated in a globe plot using the program Circos-0.69-6 (http://circos.ca) [60]. The nonsynonymous (Ka) and synonymous (Ks) substitution rates of each gene pairs were calculated using the TBtools software [58]. The Ks values were used to calculate the divergence time with the following formula: T = Ks/2λ (λ = 6.5 × 10^− 9^ for Grapevine) [61].

### Plant materials and treatments

The powdery mildew-resistant, Chinese wild *V. quinquangularis* accession ‘Shang-24’, seedless *V. vinifera* cultivars ‘Thompson Seedless’ and ‘Flame Seedless’, and seeded cultivars *V. vinifera* ‘Red Globe’ and *V. labrusca* × *V. vinifera* ‘Kyoho’ were maintained under natural environmental conditions at the grapevine germplasm resource vineyard of Northwest A&F University, Yangling, Shaanxi, China (34°20′N, 108°24′E). Young leaves of ‘Shang-24’ were inoculated with *Erysiphe necator* Schw. [syn. *Uncinula necator* (Schw.) Burr.] as previously described [62] and harvested 12, 24, 48, 72, 96, and 120 h post-inoculation. Control leaves were inoculated with sterile water. Hormone treatments were performed as a foliar spray with 300 μM ABA, 0.5 g/L Eth, 50 μM MeJA, or 100 μM SA, and samples were collected at 0.5, 1, 3, 6, 12, 24, and 48 h post-treatment [63]. Control leaves were sprayed with sterile water. Ovules were dissected from the two seedless and two seeded grapevine genotypes at 27, 30, 33, 36 and 39 days after flowering [64]. All samples were immediately frozen in liquid nitrogen and stored at −80 °C.

### RNA isolation and expression profiling

Total RNA was isolated from grapevine tissues using an EZNA Plant RNA Kit (R6827–01, Omega Bio-tek, USA). RNA quality was assessed by the ratio of A_260_/A_280_ and quantity was determined using a NanoDrop 2000 Spectrophotometer (Thermo Fisher Scientific, Wilmington, DE, USA). First-strand cDNAs were synthesized by reverse transcription of 1000 ng total DNA-free RNA using the Prime Script RT reagent Kit (TaKaRa Biotechnology, Dalian, China) following the manufacturer’s instructions. The resulting cDNA was diluted six fold for use in semi-quantitative RT-PCR and quantitative RT-PCR experiments.

Oligonucleotide primers for each *VviBBX* gene were designed using Primer Premier 5.0 software (PREMIER Biosoft International, Palo Alto, CA, USA), and assessed for potential alternative target sequences utilizing the Primer-BLAST online program in the NCBI database (https://www.ncbi.nlm.nih.gov/tools/primer-blast/index.cgi?LINK_LOC=BlastHome) (Additional file 12: Table S5). Expression profiles of *VviBBXs* in various organs and tissues based on the microarray data obtained from the NCBI gene expression omnibus (GEO) datasets under the series entry (GSE36128) (https://www.ncbi.nlm.nih.gov/geo/query/acc.cgi?acc=GSE36128) [31]. The grapevine *ACTIN1* gene (GenBank accession no. AY680701) and *EF1-α* gene (GenBank accession no. EC931777) were used as the endogenous control. Semi-quantitative RT-PCR and quantitative RT-PCR were carried out as previously described [65]. The results of semi-quantitative RT-PCR were visualized using the software GeneSnap (Version 7.08; SynGene, Cambridge, England) and heat maps were generated using TBtools software [58]. The 2^−ΔΔCT^ method was used to calculate gene relative expression levels from quantitative RT-PCR amplification [66], and StepOne software (Version 2.3; Applied BioSystems, USA) was used to analyze the relative expression.

Significant differences (*P< 0.05; **P< 0.01, respectively) between samples were determined with a *t*-test using the SPSS 25.0 software package (SPSS Inc., Chicago, IL, USA), and illustrated using Sigmaplot version 14.0 (Systat software, Inc., CA, USA).

### Subcellular localization and transcriptional activity of BBX proteins

The subcellular location of BBX proteins was predicted by CELLO (http://cello.life.nctu.edu.tw) [67]. The full-length of coding sequences of three *BBX* genes were amplified with high fidelity PrimeSTAR^®^ Max DNA Polymerase (TaKaRa Biotechnology, Dalian, China) from ‘Thompson Seedless’ (*VviBBX8*) and ‘Shang-24’ (*VquBBX15a* and *VquBBX29b*). The three grapevine *BBX* coding regions without the termination codon were inserted into a pCambia2300-GFP vector driven by the CaMV 35S promoter. The resulting constructs were transformed into *Agrobacterium tumefaciens* GV3101 and infected into the leaves of *Nicotiana benthamiana* as previously described [68]. The transient expression of the fused proteins was observed by a laser scanning confocal microscope (Olympus FV3000, Japan) after 3 d at room temperature. The green fluorescence and DAPI (4,6-diamidino-2-phenylindole dihydrochloride) were excited with a 488-nm and 405-nm laser line, respectively.

Transcriptional activity assays were performed using the Matchmaker^®^ Gold Yeast Two-Hybrid System (Clontech, Mountain View, CA, USA) as described in the manual. Full-length *BBXs* were fused in the pGBKT7 vector containing the DNA-binding region of GAL4, and transferred into the yeast strain Y2H. Meanwhile, pGBKT7-53 co-transformed with pGADT7-T was used as positive control, and pGBKT7-Lam co-transformed with pGADT7-T was used as negative control. The transformants were grown at 30 °C for 3–5 days. Transcriptional activation activity was determined by positive blue colonies on the selective solid medium plate SD/−Trp, supplemented with 40 μg/mL X-α-Gal and 200 ng/mL AbA. Primers used for gene clone, subcellular localization and transcriptional activity were listed in Additional file 13: Table S6.

## Supplementary Information

**Additional file 1: Text S1.** The CDS sequences of *BBX* family in grapevine

**Additional file 2: Text S2.** The protein sequences of BBX family in six species

**Additional file 3: Fig. S1.** Alignment of the conserved domains of grapevine BBX proteins

**Additional file 4: Fig. S2.** The differences of B-box2 domains of grapevine and *Arabidopsis* BBX members in Group I and Group II

**Additional file 5: Table S1.** The motif sequences of BBX proteins identified by MEME tools

**Additional file 6: Table S2.** Segmental duplications within grapevine *VviBBX* genes and Ka/Ks ratios analysis of segmental duplicate gene pairs

**Additional file 7: Table S3.** Segmental duplications of *BBX* genes between grapevine and *Arabidopsis* and Ka/Ks ratios analysis of segmental duplicate gene pairs

**Additional file 8: Fig. S3.** Tissue-specific expression analysis of grapevine *VviBBX* genes. Berry Pericarp (−FS: fruit set, −PFS: post-fruit set, −V: véraison, −MR: mid-ripening, −R: ripening); Bud (−S: swell, −B: burst, −AB: after-burst, −L: latent bud, −W: winter bud); Berry Flesh (−PHWI: post-harvest withering I, −PHWII: post-harvest withering II, −PHWIII: post-harvest withering III); Inflorescence (−Y: young inflorescence, −WD: well developed inflorescence); Flower (−FB: flowering begins, −F: flowering); Leaf (−FS: mature leaf, −S: senescencing leaf); Stem (−G: green stem, −W: woody stem)

**Additional file 9: Table S4.** The microarray data of the grapevine *VviBBX* genes in different organs, tissues and developmental stages

**Additional file 10: Fig. S4.** Alignment of the coding sequences of three cloned *BBX* genes

**Additional file 11: Fig. S5.** The number of BBX proteins in other species

**Additional file 12: Table S5.** Primer sequences used in expression analysis of *BBX* genes in grapevine

**Additional file 13: Table S6.** Primers used for gene clone, subcellular localization and transcriptional activity

## Data Availability

The grapevine reference genome assembly (12X.v2) and its VCost.v3 gene annotation, as well as the *VviBBX* coding sequences and protein sequences, are available URIG website (https://urgi.versailles.inra.fr/Species/Vitis/Annotations) [47]. The B-box-type zinc finger domain HMM (Hidden Markov Model) profile numbered PF00643 was extracted from the Pfam database (http://pfam.xfam.org/family/PF00643) [48]. The CRIBI v2.1 ID and Locus ID were obtained from Phytozome v13 database (https://phytozome-next.jgi.doe.gov) [50] and Grape Genome Browser (12X) database (https://www.genoscope.cns.fr/vitis/) [26], respectively. The accession numbers of VviBBX proteins in Table 1 are retrieved from NCBI repository (https://www.ncbi.nlm.nih.gov/). The Arabidopsis, pear, rice, tomato and apple BBX protein sequences were downloaded from the Arabidopsis Information Resource (TAIR) (https://www.arabidopsis.org), GigaDB database (http://gigadb.org/site/index), Rice Genome Annotation Project (http://rice.plantbiology.msu.edu), Solanaceae Genomics Network (https://solgenomics.net) and Genome Database for Rosaceae (https://www.rosaceae.org), respectively. The microarray data for expression profiles in various organs and tissues at different developmental stages are available on NCBI GEO under the accession number GSE36128 (https://www.ncbi.nlm.nih.gov/geo/query/acc.cgi?acc=GSE36128) [31]. The endogenous control genes are retrieved from NCBI accession numbers as AY680701 (*ACTIN1*; https://www.ncbi.nlm.nih.gov/nuccore/AY680701.1/) and EC931777 (*EF1-α*; https://www.ncbi.nlm.nih.gov/nuccore/EC931777.1/).

## Abbreviations

TFs: Transcription factors
BBX: B-box
HMM: Hidden Markov Model
SMART: Simple Modular Architecture Research Tool
CDD: Conserved Domain Database
Ka: The rate of nonsynonymous substitutions
Ks: The rate of synonymous substitutions
Mya: Million years ago
ABA: Abscisic acid
Eth: Ethylene
MeJA: Methyl jasmonate
SA: Salicylic acid
RT-PCR: Real-time polymerase chain reaction
NJ: Neighbor-Joining
DAF: Days after flowering
DAPI: 4,6-diamidino-2-phenylindole dihydrochloride
Y2H: Yeast Two-Hybrid System
SD/−Trp: Lacking tryptophan
X-α-Gal: 5-Bromo-4-chloro-3-indolyl-α-Dgalactopyranoside
AbA: Aureobasidin A
