# Supplementary material for: Genome-wide identification and expression analysis of the B-box transcription factor gene family in grapevine (Vitis vinifera L.)
Source: BMC Genomics. 2021 Mar 29;22:221. doi: 10.1186/s12864-021-07479-4 (PMC8008696; doi:10.1186/s12864-021-07479-4)
Supplement: Supplementary file 3 — Additional file 3: Fig. S1. Alignment of the conserved domains of grapevine BBX proteins [file 12864_2021_7479_MOESM3_ESM.pdf]

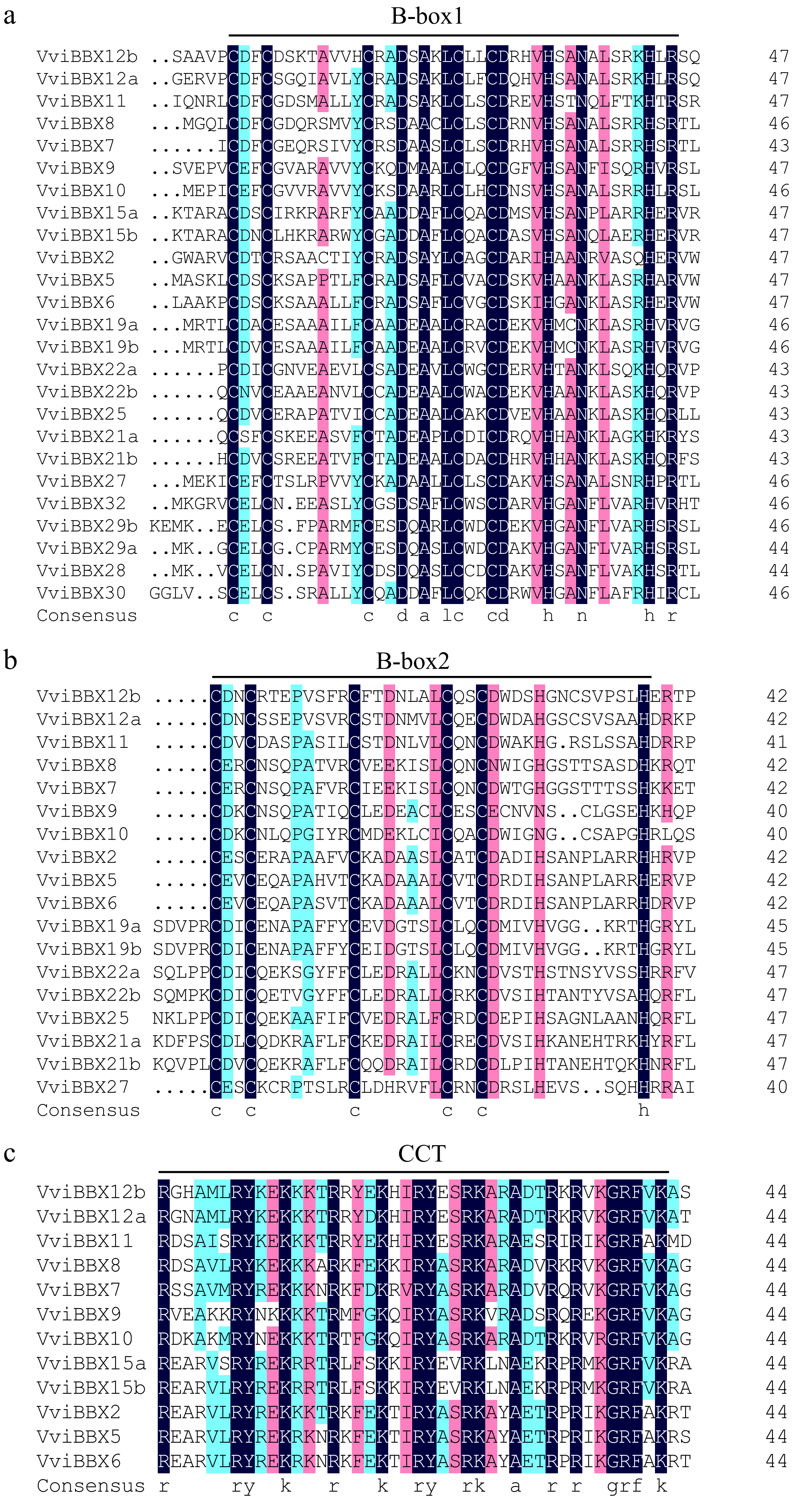
a
B-box1
VviBBX12b ..SAAVPCDFCDSKTAVVHCRADSAKLCLLCDRHVHSANALSRKHLRSQ 47
VviBBX12a ..GERVPCDFCSGQIAVLYCRADSAKLCLFCDQHVHSANALSRKHLRSQ 47
VviBBX11 ..IQNRLCDFCGDSMALLYCRADSAKLCLSCDREVHSTNQLFTKHTRSR 47
VviBBX8 ...MGQLCDFCGDQRSMVYCRSDAACLCLSCDRNVHSANALSRRHSRTL 46
VviBBX7 ......ICDFCGEQRSIVYCRSDAASLCLSCDRHVHSANALSRRHSRTL 43
VviBBX9 ..SVEPVCEFCGVARAVVYCKQDMAALCLQCDGFVHSANFISQRHVRSL 47
VviBBX10 ...MEPICEFCGVVRAVVYCKSDAARLCLHCDNSVHSANALSRRHLRSL 46
VviBBX15a ..KTARACDSCIRKRARFYCAADDAFLCQACDMSVHSANPLARRHERVR 47
VviBBX15b ..KTARACDNCLHKRARWYCGADDAFLCQACDASVHSANQLAERHERVR 47
VviBBX2 ..GWARVCDTCRSAACTIYCRADSAYLCAGCDARIHAANRVASQHERVW 47
VviBBX5 ..MASKLCDSCKSAPPTLFCRADSAFLCVACDSKVHAANKLASRHARVW 47
VviBBX6 ..LAAKPCDSCKSAAALLFCRADSAFLCVGCDSKIHGANKLASRHERVW 47
VviBBX19a ...MRTLCDACESAAAILFCAADEAALCRACDEKVHMCNKLASRHVRVG 46
VviBBX19b ...MRTLCDVCESAAAILFCAADEAALCRVCDEKVHMCNKLASRHVRVG 46
VviBBX22a ......PCDICGNVEAEVLCSADEAVLCWGCDERVHTANKLSQKHQRVP 43
VviBBX22b ......QCNVCEAAEANVLCCADEAALCWACDEKVHAANKLASKHQRVP 43
VviBBX25 ......QCDVCERAPATVICCADEAALCAKCDVEVHAANKLASKHQRLL 43
VviBBX21a ......QCSFCSKEEASVFCTADEAPLCDICDRQVHHANKLAGKHKRYS 43
VviBBX21b ......HCDVCSREEATVFCTADEAALCDACDHRVHHANKLASKHQRFS 43
VviBBX27 ...MEKICEFCTSLRPVVYCKADAALLCLSCDAKVHSANALSNRHPRTL 46
VviBBX32 ..MKGRVCELCN.EEASLYCGSDSAFLCWSCDARVHGANFLVARHVRHT 46
VviBBX29b KEMK..ECELCS.FPARMFCESDQARLCWDCDEKVHGANFLVARHSRSL 46
VviBBX29a ..MK..GCELCG.CPARMYCESDQASLCWDCDAKVHGANFLVARHSRSL 44
VviBBX28 ..MK..VCELCN.SPAVIYCDSDQASLCCDCDAKVHSANFLVAKHSRTL 44
VviBBX30 GGLV..SCELCS.SRALLYCQADDAFLCQKCDRWVHGANFLAFRHIRCL 46
Consensus c c c d a lc cd h n h r
b
B-box2
VviBBX12b .....CDNCRTEPVSFRCFTDNLALCQSCDWDSHGNCSVPSLHERTP 42
VviBBX12a .....CDNCSSEPVSVRCSTDNMVLCQECDWDAHGSCSVSAAHDRKP 42
VviBBX11 .....CDVCDASPASILCSTDNLVLCQNCDWAKHG.RSLSSAHDRRP 41
VviBBX8 .....CERCNSQPATVRCVEEKISLCQNCNWIGHGSTTSASDHKRQT 42
VviBBX7 .....CERCNSQPAFVRCIEEKISLCQNCDWTGHGGSTTTSSHKKET 42
VviBBX9 .....CDKCNSQPATIQCLEDEACLCESCECNVNS..CLGSEHKHQP 40
VviBBX10 .....CDKCNLQPGIYRCMDEKLCICQACDWIGNG..CSAPGHRLQS 40
VviBBX2 .....CESCERAPAAFVCKADAASLCATCDADIHSANPLARRHHRVP 42
VviBBX5 .....CEVCEQAPAHVTCKADAAALCVTCDRDIHSANPLARRHERVP 42
VviBBX6 .....CEVCEQAPASVTCKADAAALCVTCDRDIHSANPLARRHDRVP 42
VviBBX19a SDVPRCDICENAPAFFYCEVDGTSLCLQCDMIVHVGG..KRTHGRYL 45
VviBBX19b SDVPRCDICENAPAFFYCEIDGTSLCLQCDMIVHVGG..KRTHGRYL 45
VviBBX22a SQLPPCDICQEKSGYFFCLEDRALLCKNCDVSTHSTNSYVSSHRRFV 47
VviBBX22b SQMPKCDICQETVGYFFCLEDRALLCRKCDVSIHTANTYVSAHQRFL 47
VviBBX25 NKLPPCDICQEKAAFIFCVEDRALFCRDCDEPIHSAGNLAANHQRFL 47
VviBBX21a KDFPSCDLCQDKRAFLFCKEDRAILCRECDVSIHKANEHTRKHYRFL 47
VviBBX21b KQVPLCDVCQEKRAFLFCQQDRAILCRDCDLPIHTANEHTQKHNRFL 47
VviBBX27 .....CESCKCRPTSLRCLDHRVFLCRNCDRSLHEVS..SQHHRRAI 40
Consensus c c c c c h
c
CCT
VviBBX12b RGHAMLRYKEKKKTRRYEKHIRYESRKARADTRKRVKGRFVKAS 44
VviBBX12a RGNAMLRYKEKKKTRRYDKHIRYESRKARADTRKRVKGRFVKAT 44
VviBBX11 RDSAISRYKEKKKTRRYEKHIRYESRKARAESRIRIKGRFAKMD 44
VviBBX8 RDSAVLRYKEKKKARKFEKKIRYASRKARADVRKRVKGRFVKAG 44
VviBBX7 RSSAVMRYREKKKNRKFDKRVRYASRKARADVRQRVKGRFVKAG 44
VviBBX9 RVEAKKRYNKKKKTRMFGKQIRYASRKVRADSRQREKGRFVKAG 44
VviBBX10 RDKAKMRYNEKKKTRTFGKQIRYASRKARADTRKRVRGRFVKAG 44
VviBBX15a REARVSRYREKRRTRLFSKKIRYEVRKLNAEKRPRMKGRFVKRA 44
VviBBX15b REARVLRYREKRRTRLFSKKIRYEVRKLNAEKRPRMKGRFVKRA 44
VviBBX2 REARVLRYREKKKTRKFEKTIRYASRKAYAETRPRIKGRFAKRT 44
VviBBX5 REARVLRYREKRKNRKFEKTIRYASRKAYAETRPRIKGRFAKRS 44
VviBBX6 REARVLRYREKRKNRKFEKTIRYASRKAYAETRPRIKGRFAKRT 44
Consensus r ry k r k ry rk a r r grf k
