# Supplementary material for: Genome-wide identification and expression analysis of the B-box transcription factor gene family in grapevine (Vitis vinifera L.)
Source: BMC Genomics. 2021 Mar 29;22:221. doi: 10.1186/s12864-021-07479-4 (PMC8008696; doi:10.1186/s12864-021-07479-4)
Supplement: Supplementary file 4 — Additional file 4: Fig. S2. The differences of B-box2 domains of grapevine and Arabidopsis BBX members in Group I and Group II [file 12864_2021_7479_MOESM4_ESM.pdf]

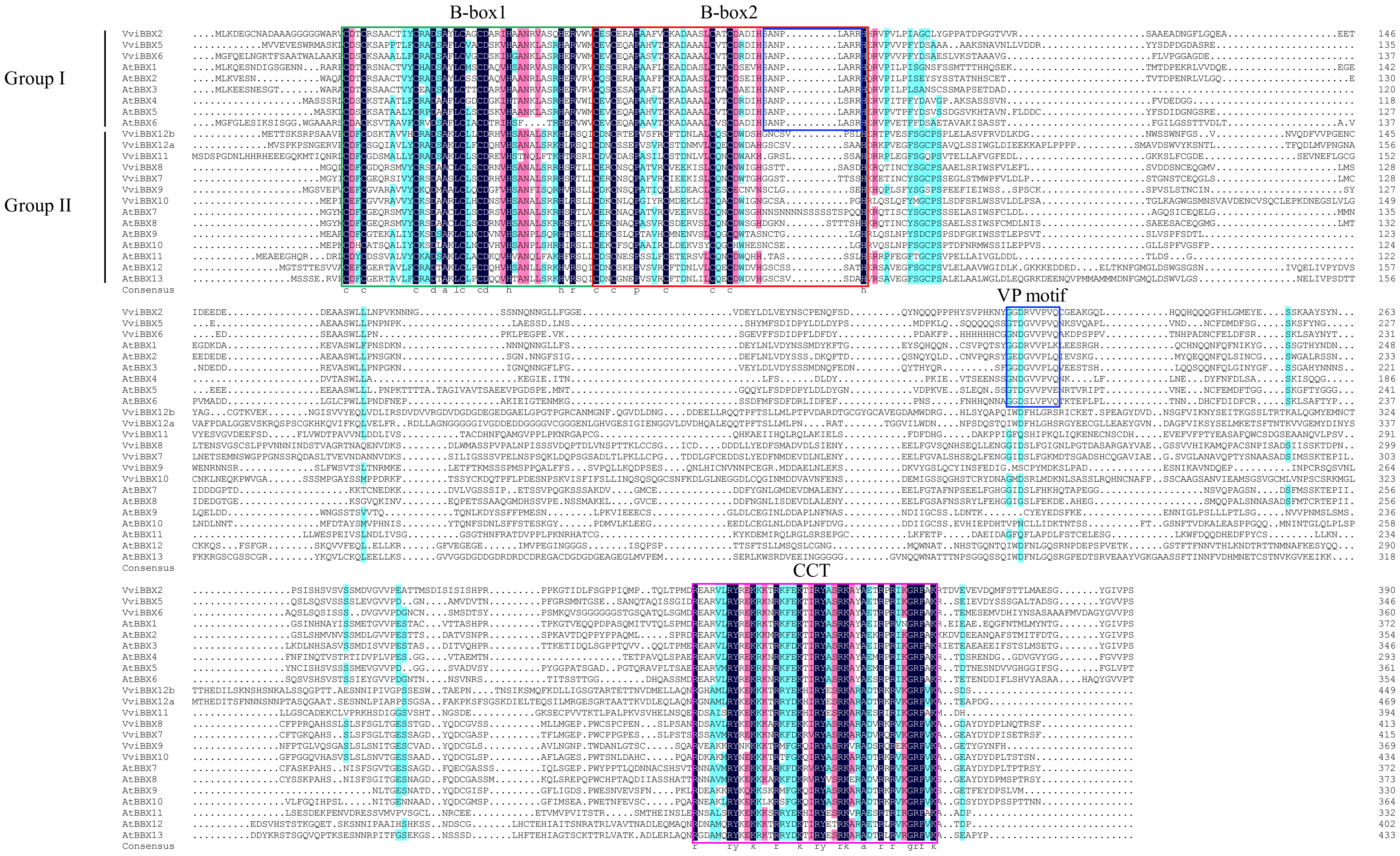
B-box1
B-box2
VP motif
CCT
Group I
Group II
VviBBX2
VviBBX5
VviBBX6
AtBBX1
AtBBX2
AtBBX3
AtBBX4
AtBBX5
AtBBX6
VviBBX12b
VviBBX12a
VviBBX11
VviBBX8
VviBBX7
VviBBX9
VviBBX10
AtBBX7
AtBBX8
AtBBX9
AtBBX10
AtBBX11
AtBBX12
AtBBX13
Consensus
