# Supplementary material for: Genome-wide identification and expression analysis of the B-box transcription factor gene family in grapevine (Vitis vinifera L.)
Source: BMC Genomics. 2021 Mar 29;22:221. doi: 10.1186/s12864-021-07479-4 (PMC8008696; doi:10.1186/s12864-021-07479-4)
Supplement: Supplementary file 5 — Additional file 5: Table S1. The motif sequences of BBX proteins identified by MEME tools [file 12864_2021_7479_MOESM5_ESM.docx]

**Table S1** The motif sequences of BBX proteins identified by MEME tools

| Name | Logo | Sequences | E-value | Sites | Width |
| --- | --- | --- | --- | --- | --- |
| Motif1 | 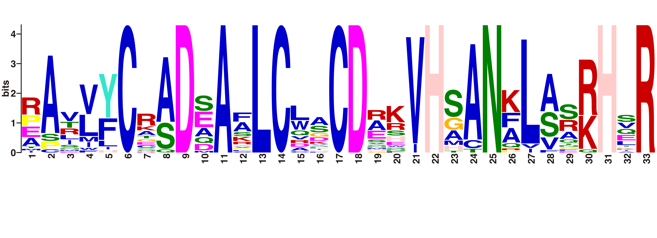 | RAVVYCRADEAALCLACDAKVHSANKLASRHSR | 1.8e-406 | 25 | 33 |
| Motif2 | 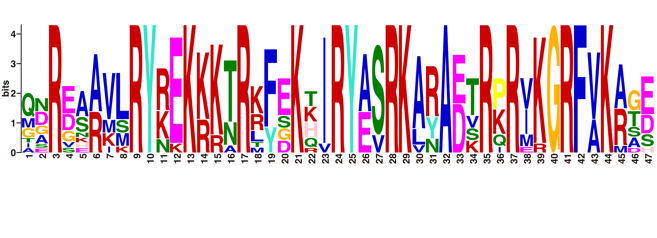 | QNREAAVLRYREKKKTRKFEKKIRYASRKARAETRPRVKGRFVKAGE | 1.5e-294 | 12 | 47 |
| Motif3 | 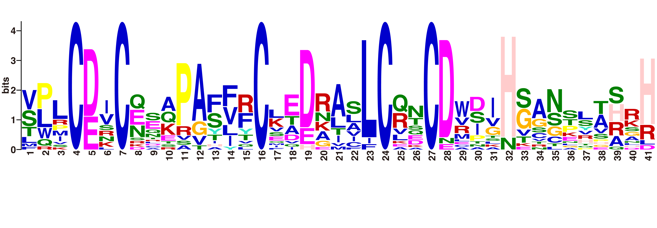 | VPLCDICEEAPAFFRCLEDRASLCQNCDWDIHSANSLASRH | 8.5e-199 | 18 | 41 |
| Motif4 | 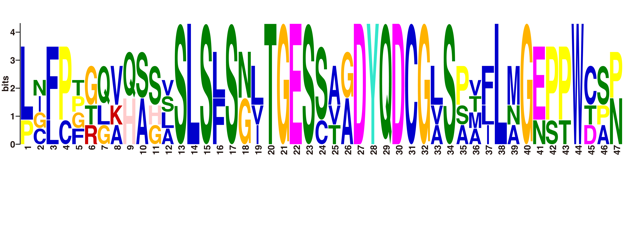 | LGFPPGQVQASASLSFSGJTGESSAGDYQDCGLSPVFLMGEPPWCSP | 1.0e-028 | 4 | 47 |
| Motif5 | 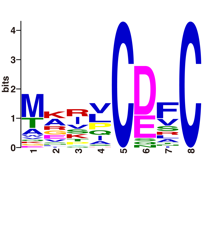 | MKRVCDFC | 1.6e-025 | 25 | 8 |
| Motif6 | 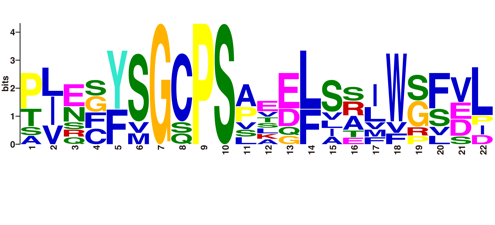 | PIESYSGCPSAEELSSIWGFEL | 6.4e-018 | 8 | 22 |
| Motif7 | 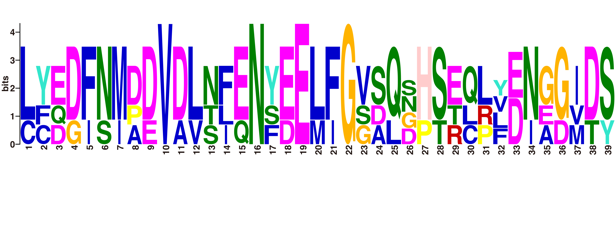 | LYEDFNMDDVDLNFENYEELFGVSQNHSEQLFDNGGIDS | 9.1e-015 | 4 | 39 |
| Motif8 | 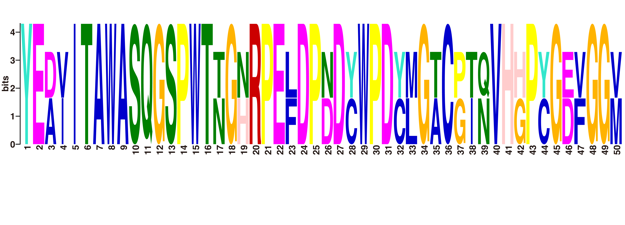 | YEDIITAWASQGSPWTNGNRPEFDPDDCWPDCLGACGINVHGPCGEFGGM | 1.6e-012 | 2 | 50 |
| Motif9 | 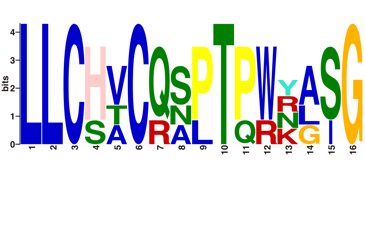 | LLCHVCQSPTPWKASG | 3.9e-008 | 4 | 16 |
| Motif10 | 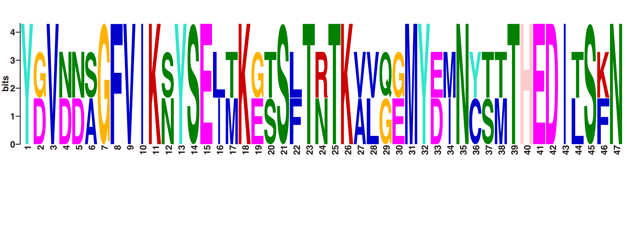 | YGVDDAGFVIKNYSEJMKESSFTRTKVLGEMYEINCSMTHEDILSFN | 1.0e-007 | 2 | 47 |
| Motif11 | 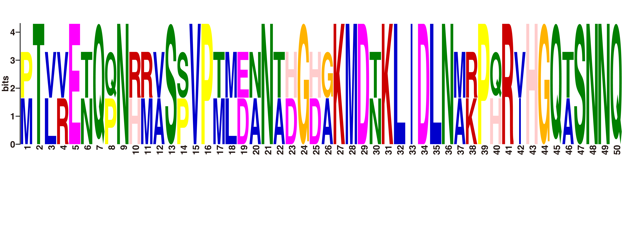 | PTLRENQPNRRVSPVPMLENNADGDGKMDNKLIDLNAKPQRIHGQASNNQ | 2.2e-006 | 2 | 50 |
| Motif12 | 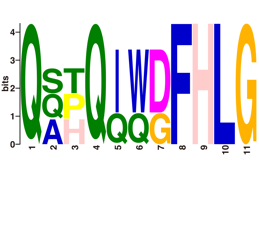 | QAPQIWDFHLG | 3.5e-004 | 3 | 11 |
| Motif13 | 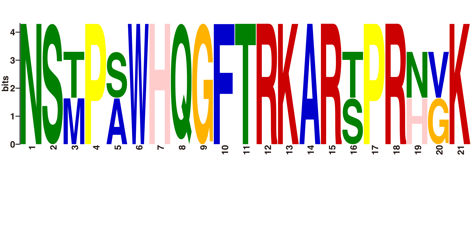 | NSMPAWHQGFTRKARSPRNGK | 9.2e-004 | 2 | 21 |
| Motif14 | 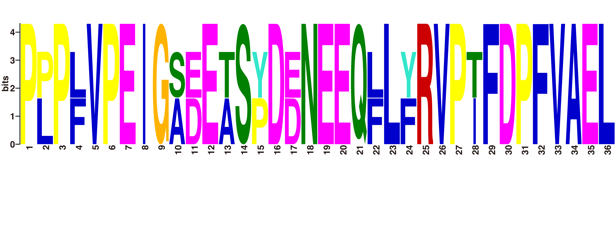 | PPPFVPEIGAEEASPDENEEQFLFRVPIFDPFVAEL | 2.3e-003 | 2 | 36 |
| Motif15 | 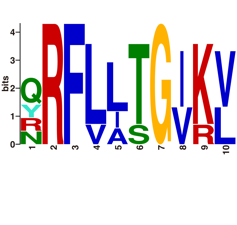 | QRFLLTGIKV | 4.1e-003 | 5 | 10 |
| Motif16 | 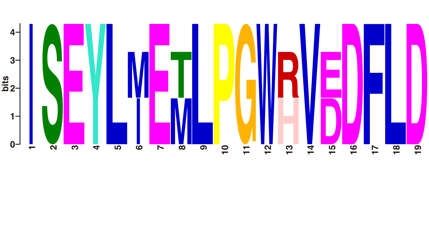 | ISEYLIEMLPGWRVEDFLD | 1.1e-002 | 2 | 19 |
