# Supplementary figures and images for: Genome-wide identification and expression analysis of the B-box transcription factor gene family in grapevine (Vitis vinifera L.)
Source: BMC Genomics. 2021 Mar 29;22:221. doi: 10.1186/s12864-021-07479-4 (PMC8008696; doi:10.1186/s12864-021-07479-4)

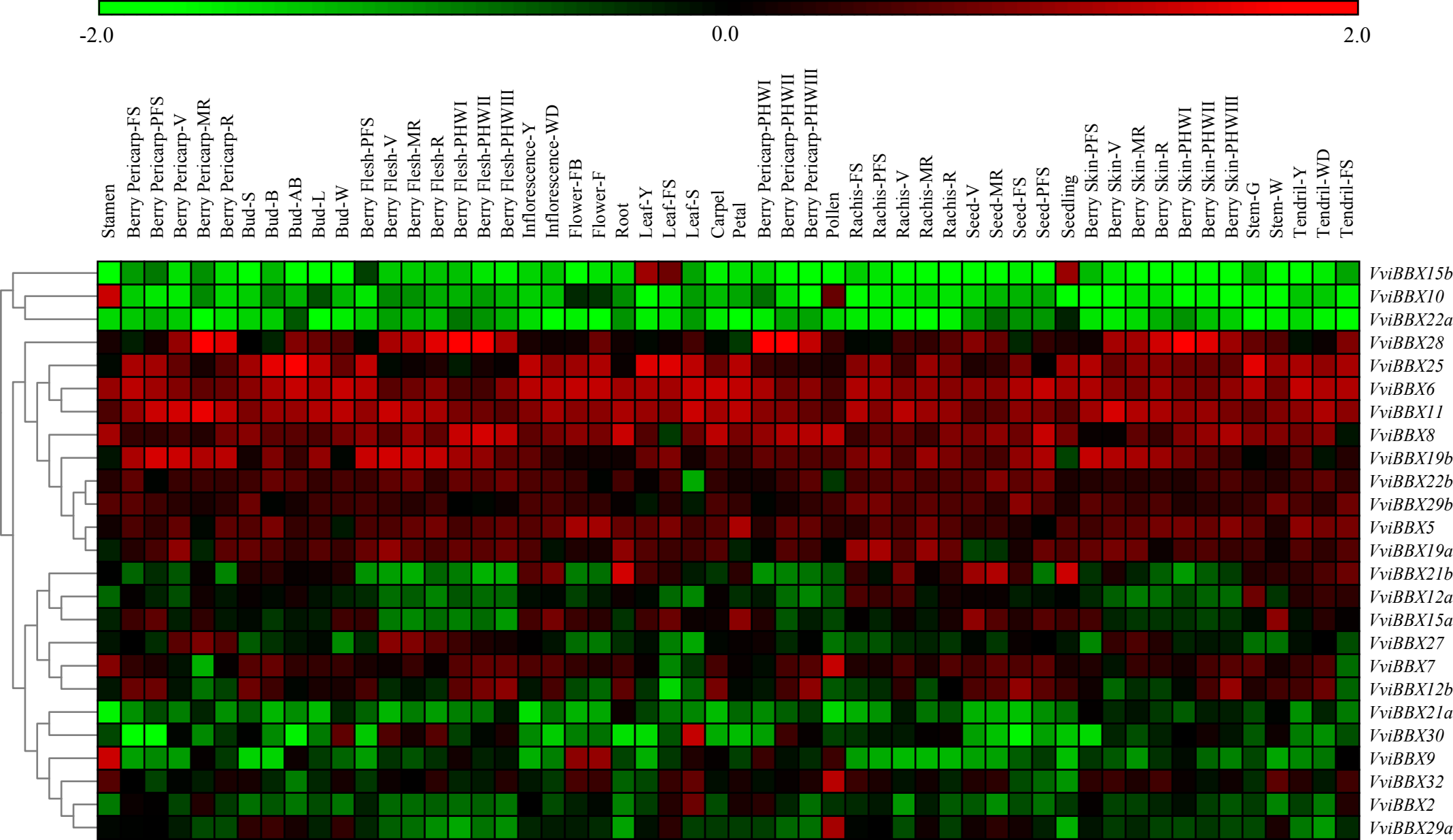

Supplement: Supplementary file 8 — Additional file 8: Fig. S3. Tissue-specific expression analysis of grapevine VviBBX genes. Berry Pericarp (−FS: fruit set, −PFS: post-fruit set, −V: véraison, −MR: mid-ripening, −R: ripening); Bud (−S: swell, −B: burst, −AB: after-burst, −L: latent bud, −W: winter bud); Berry Flesh (−PHWI: post-harvest withering I, −PHWII: post-harvest withering II, −PHWIII: post-harvest withering III); Inflorescence (−Y: young inflorescence, −WD: well developed inflorescence); Flower (−FB: flowering begins, −F: flowering); Leaf (−FS: mature leaf, −S: senescencing leaf); Stem (−G: green stem, −W: woody stem) [file 12864_2021_7479_MOESM8_ESM.pdf]

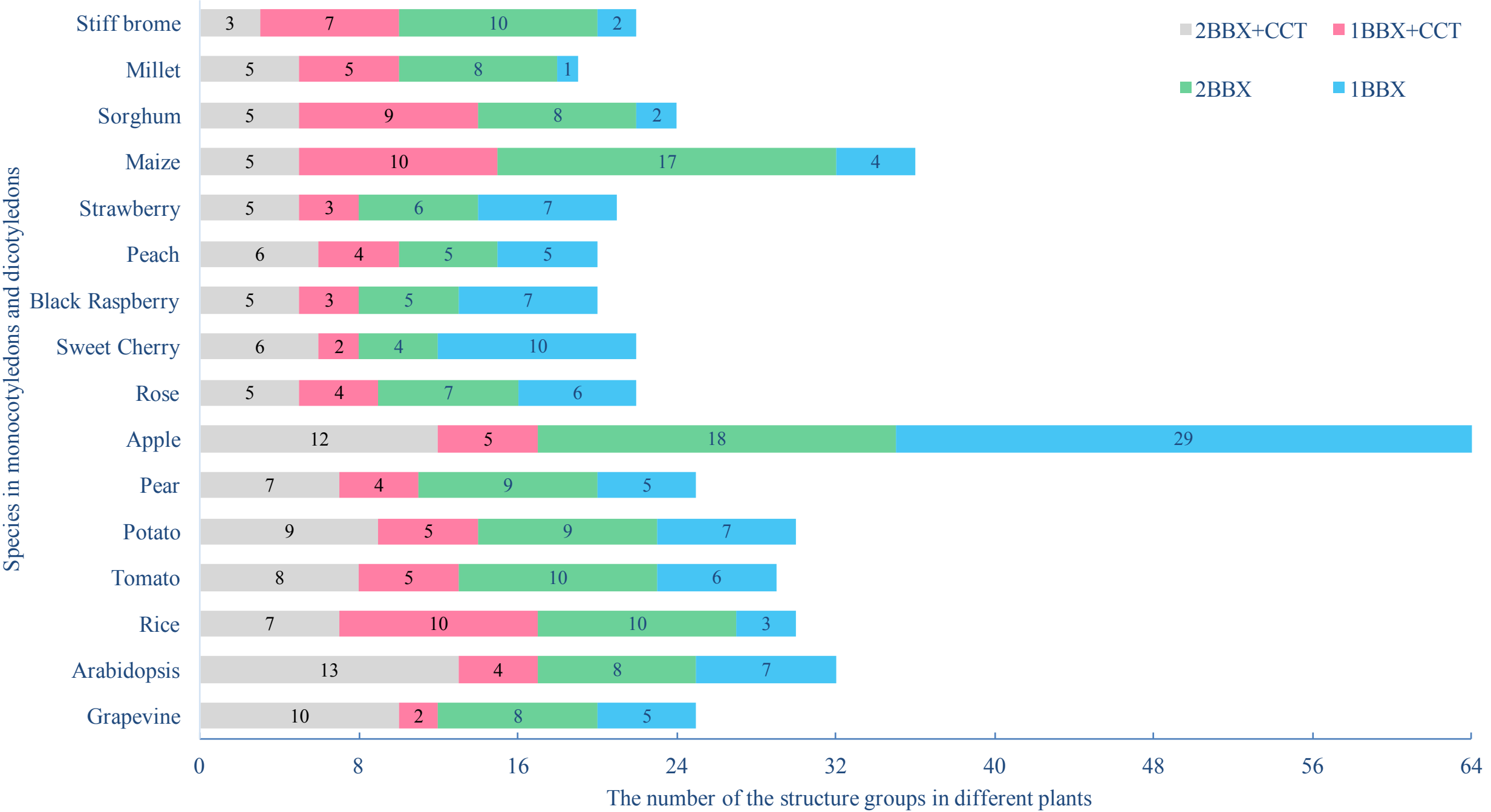

Supplement: Supplementary file 11 — Additional file 11: Fig. S5. The number of BBX proteins in other species [file 12864_2021_7479_MOESM11_ESM.pdf]
