# Supplementary material for: Genome-wide identification and expression analysis of the B-box transcription factor gene family in grapevine (Vitis vinifera L.)
Source: BMC Genomics. 2021 Mar 29;22:221. doi: 10.1186/s12864-021-07479-4 (PMC8008696; doi:10.1186/s12864-021-07479-4)
Supplement: Supplementary file 10 — Additional file 10: Fig. S4. Alignment of the coding sequences of three cloned BBX genes [file 12864_2021_7479_MOESM10_ESM.pdf]

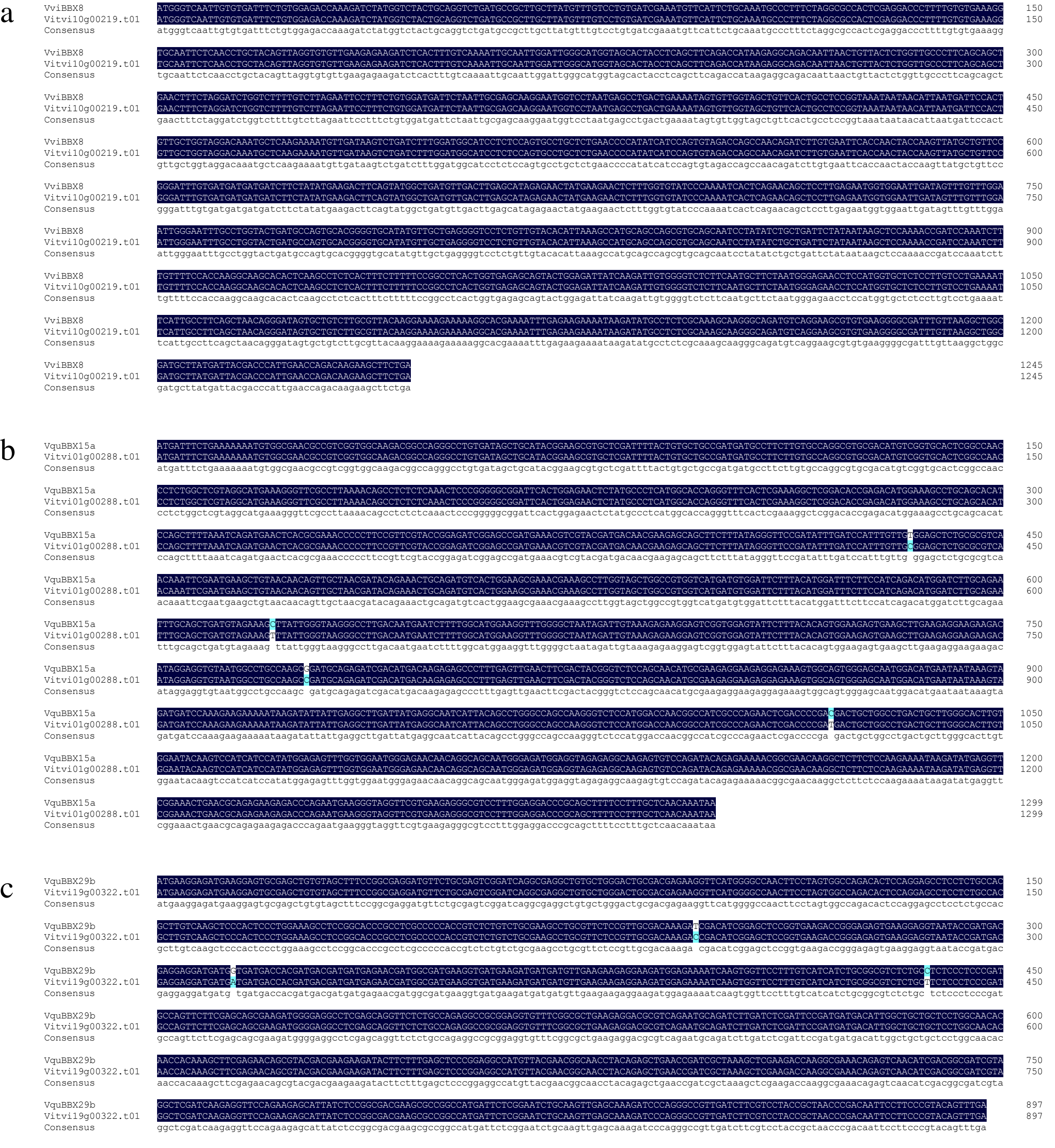

a
VviBBX8
Vitvi10g00219.t01
Consensus
150
300
450
600
750
900
1050
1200
1245
b
VquBBX15a
Vitvi01g00288.t01
Consensus
150
300
450
600
750
900
1050
1200
1299
c
VquBBX29b
Vitvi19g00322.t01
Consensus
150
300
450
600
750
897
